# Occurrence of carbapenem-resistant hypervirulent *Klebsiella pneumoniae* in oysters in Egypt: a significant public health issue

**DOI:** 10.1186/s12941-024-00711-5

**Published:** 2024-06-17

**Authors:** Rahma Mohammed, Sara M. Nader, Dalia A. Hamza, Maha A. Sabry

**Affiliations:** Department of Zoonoses, Faculty of Veterinary Medicine, Cairo University, PO Box 12211, Giza, Egypt

**Keywords:** *Klebsiella pneumoniae*, Carbapenem-resistant hypervirulent *K. pneumoniae*, Oysters, Egypt

## Abstract

**Background:**

The global dissemination of critical-priority carbapenem-resistant hypervirulent *Klebsiella pneumoniae* (CR-hvKp) via food sources represents a significant public health concern. Epidemiological data on CR-hvKp in oysters in Egypt is limited. This study aimed to investigate the potential role of oysters sold in Egypt as a source for carbapenem-resistant *K. pneumoniae* (CRKP), hypervirulent *K. pneumoniae* (hvKp), and CR-hvKp and assess associated zoonotic risks.

**Methods:**

A sample of 330 fresh oysters was randomly purchased from various retail fish markets in Egypt and divided into 33 pools. Bacteriological examination and the identification of *Klebsiella pneumoniae* were performed. Carbapenem resistance in *K. pneumoniae* isolates was determined by phenotypic and molecular methods. Additionally, the presence of hypervirulent *K. pneumoniae* was identified based on virulence gene markers (*peg-344*, *rmpA*, *rmpA2*, *iucA*, and *iroB*), followed by a string test. The clustering of CR-hvKp strains was carried out using R with the pheatmap package.

**Results:**

The overall prevalence of *K. pneumoniae* was 48.5% (16 out of 33), with 13 isolates displaying carbapenem resistance, one intermediate resistance, and two sensitive. Both carbapenem-resistant *K. pneumoniae* and carbapenem-intermediate-resistant *K. pneumoniae* strains exhibited carbapenemase production, predominantly linked to the *bla*_VIM_ gene (68.8%). HvKp strains were identified at a rate of 62.5% (10/16); notably, *peg-344* was the most prevalent gene. Significantly, 10 of the 13 CRKP isolates possessed hypervirulence genes, contributing to the emergence of CR-hvKp. Moreover, cluster analysis revealed the clustering of two CR-hvKp isolates from the same retail fish market.

**Conclusion:**

This study provides the first insight into the emergence of CR-hvKp among oysters in Egypt. It underscores the potential role of oysters as a source for disseminating CR-hvKp within aquatic ecosystems, presenting a possible threat to public health.

## Introduction

The worldwide spread of antimicrobial resistance poses a significant threat to public health, as highlighted by the World Health Organization [1]. *Klebsiella pneumoniae* is recognized as a high-priority pathogen due to its resistance to antimicrobials, which necessitates developing novel control strategies [2, 3]. This bacterium is an opportunistic, Gram-negative pathogen from the *Enterobacteriaceae* family [4]. It is abundant in the environment and can be found in the gastrointestinal tract of healthy humans and animals [5].

*K. pneumoniae* can be categorized into classic *Klebsiella pneumoniae* (cKp) and hypervirulent *Klebsiella pneumoniae* (hvKp), both of which provide unique challenges to clinicians [6]. The classical strain infects immunocompromised individuals, causing pneumonia, urinary tract, and wound infections. HvKp, an emerging pathogen, exhibits greater virulence compared to cKp. It is distinguished by its community-acquired nature, leading to severe invasive infections with metastatic characteristics. These infections commonly manifest as pyogenic liver abscesses, endophthalmitis, meningitis, and skin and soft tissue infections, particularly affecting immunocompetent individuals [7, 8]. HvKp and cKp are worldwide pathogens, but in the last decades, the incidence of hvKp has increased in Egypt and worldwide [9, 10].

Recently, carbapenem-resistant hypervirulent *Klebsiella pneumoniae* (CR-hvKp) has emerged as a significant health threat to human health [11]. This strain of *Klebsiella pneumoniae* not only possesses resistance to carbapenems, commonly used as a last line of treatment against bacterial infections, but it is also highly virulent and capable of causing severe infections even in healthy individuals [12].

One of the mechanisms responsible for the evolution of CR-hvKp is the acquisition of a hybrid plasmid carrying both the hypervirulence genes and the carbapenemase genes [13].

Carbapenemases such as *K. pneumoniae* carbapenemases (KPC), metallo-β-lactamases (MBL), including Verona integron-encoded metallo-beta-lactamase (VIM) and New Delhi metallo-beta-lactamase (NDM) types, and oxacillin-hydrolyzing metallo-β-lactamases (OXA-48) are primary mechanisms by which *K. pneumoniae* resist carbapenem antibiotics [14].

Meanwhile, the most reliable hypervirulence genes are putative transporter (*peg-344*), salmochelin siderophore biosynthesis (*iroB*), aerobactin siderophore biosynthesis (*iucA*), regulators of the mucoid phenotype (*rmpA* and *rmpA2*) [15]. These hypervirulence genes of hvKp play crucial roles in the pathogenesis of the bacterium; *peg-344* encodes for a putative transporter protein that may aid in acquiring nutrients or secretion of toxins [16]. On the other hand, *iroB* and *iucA* genes capture iron and deliver it to the bacteria, respectively [17]. In addition, *rmpA* and *rmpA2* genes regulate the expression of the capsule polysaccharide, which is a critical factor in the resistance against phagocytosis and mucoviscosity [18].

In recent years, there have been many reports of CR-hvKp infections in many parts of the world, including Asia, Europe, North America, and Africa [19–22].

The food chain can provide a pathway for humans to acquire antimicrobial resistance and virulence genes. Cross-contamination during food preparation further spreads these genes to various surfaces and utensils. Contaminated seafood may release these genes into the environment, potentially impacting human health [23, 24].

Marine bivalves, such as clams and oysters, are a valuable human food source and an essential part of the aquaculture industry, which has expanded dramatically in the last two decades [25]. These bivalves act as ecological bioindicators by filtering particles from the water that may harbor harmful bacteria [26, 27]. Accordingly, raw consumption of oysters and other bivalves poses a health hazard to consumers and remains a primary cause of seafood-related food poisoning outbreaks worldwide [28].

*Klebsiella pneumoniae* is recognized as a potentially harmful bacteria found in oysters, as indicated by studies [26, 27]. However, the exact number of cases attributed to *Klebsiella pneumoniae* remains uncertain. A recent study by Freire et al. [29] identified the presence of carbapenemase-producing *Enterobacteriaceae* (CRE), including *Klebsiella pneumoniae*, in Bivalves harvested from Portugal. It is crucial to note that this study did not specifically focus on examining the presence of hypervirulent strains of *Klebsiella pneumoniae.*

In response to this emerging problem, more research is needed to understand the potential risk of CR-hvKp in oysters and other seafood and develop effective strategies to protect public health. Therefore, the present study aimed to investigate the role of oysters as a source of CR-hvKp. Specifically, we determined the prevalence of CRKP, hvKp, and CR-hvKp in oyster samples from different markets in Egypt to assess the possible zoonotic risk associated with oyster consumption.

## Methods

### Sample collection and processing

330 fresh Bivalve mollusks (specifically oysters) were randomly purchased from different retail fish markets in Egypt over 1 year, from December 2021 to December 2022, and were immediately shipped to the laboratory under sterile refrigerated conditions. The samples were divided into 33 pools, each containing 10 oyster samples. Each pool corresponds to a distinct market, emphasizing one per market [29].

The external valves of oysters were thoroughly washed with sterile water and aseptically opened, and the digestive tissues were dissected, cleaned, and finely chopped to a paste-like consistency to ensure the uniformity of the starting material [30].

### Isolation and identification of *K. pneumoniae*

Aliquots of 2 g from each pool were enriched in Brain heart infusion broth (Oxoid, Hampshire, UK) for 24 h at 37 °C. The enrichment was streaked onto MacConkey agar (Oxoid, Hampshire, UK) and incubated at 37 °C for 24 h. Suspected *Klebsiella* spp colonies were sub-cultured to obtain a pure culture and were examined for colony morphology and phenotypic traits according to Abd-Elmonsef et al. [31].

### Antimicrobial susceptibility testing

All confirmed *K. pneumoniae* isolates were examined for sensitivity to the carbapenem antibiotics group: meropenem (10 µg) and ertapenem (10 µg) (Oxoid, Hampshire, UK).

The selection of meropenem and ertapenem for the antibiotic sensitivity assay is according to recent studies in Egypt by Ahmed et al. [32] and Gandor et al. [33] and according to data from the China Antimicrobial Surveillance Network (CHINET) [34].

The test was carried out by Kirby-Bauer disc diffusion assay on Muller-Hinton agar (HiMedia), and the results were interpreted according to the clinical breakpoints recommended by the Clinical and Laboratory Standards Institute (CLSI) [35].

### Extraction of the genomic DNA

Genomic DNA was extracted from the phenotypic-resistant, intermediate, and sensitive *K. pneumoniae* isolates using the boiling method, according to Dashti et al. [36]. The extracted DNA was stored at -20 °C until further use.

### Molecular detection of carbapenem-resistant *K. pneumoniae* (CRKP)

Multiplex PCR was used to detect carbapenemase genes, *bla*_KPC,_ and *bla*_NDM_, as previously described by Li et al. [37]. Another uniplex PCR test targeting the *bla*_VIM_ and *bla*_OXA−48_ genes was performed according to Li et al. [38] and Dallenne et al. [39], respectively. The PCR mixtures were carried out on a total volume of 25 µl, containing 3 µl of template DNA from each isolate, 12.5 µl of EmeraldAmp MAX PCR Master Mix (Takara, Japan), and 0.5 µl of each primer (10 pmol/µl; Metabion, Germany) and completed up to 25 µl by PCR-grade water. The PCR amplicons were electrophoresed on a 1.5% agarose gel and visualized under ultraviolet light. The specific oligonucleotide primers set and the amplification conditions are displayed in Table 1. Carbapenemase-positive (*K. pneumoniae* ATCC BAA-1705) and carbapenemase-negative (*K. pneumoniae* ATCC BAA-1706) control strains were used as control.

**Table 1:** The sequence of oligonucleotide primers used for PCR amplification of the carbapenemase-encoding genes and virulence genes (*rmpA*, *rmpA2*, *iucA*, *iroB* and *peg-344*) which are molecular biomarkers for hvKp

Genes (bp)	Primer sequence (5ʹ–3ʹ)	Thermal Cycles	References
**Carbapenemase-encoding genes**			
***bla***_**KPC**_ (882 bp)	**F**:ATG TCA CTG TAT CGC CGT CT **R**: TTT TCA GAG CCT TAC TGC CC	95 ºC, 5 min; 30 cycles (94 ºC, 1 min; 55 ºC, 1 min; 72 ºC, 2 min), 72 ºC, 10 min	[37]
***bla***_**NDM**_ (621 bp)	**F**:GGT TTG GCG ATC TGG TTT TC **R**:CGG AAT GGC TCA TCA CGA TC		
***bla***_**VIM**_ (261 bp)	**F**:AGT GGT GAG TAT CCG ACAG **R**:ATG AAA GTG CGT GGA GAC	95 ºC, 5 min; 35 cycles (94 ºC, 30 s; 55ºC, 30 s; 72 ºC, 1 min), 72 ºC, 10 min	[38]
***bla***_**OXA−48**_ (283 bp)	**F**: GCTTGATCGCCCTCGATT **R**: GATTTGCTCCGTGGCCGAAA	94 ºC, 10 min; 30 cycles (94 ºC, 40 s; 60 ºC, 40 s; 72 ºC, 1 min), 72 ºC, 7 min	[39]
**Virulence-encoding genes**			
***iucA*** (239 bp)	**F**: AATCAATGGCTATTCCCGCTG **R**: CGCTTCACTTCTTTCACTGACAGG	95ºC,2 min; 25 cycles (95 ºC,30 s;59 ºC,30 s; 72 ºC,30 s),72 ºC,10 min	[15, 40]
***iroB*** (585 bp)	**F**: CAAAAAAGCAGCAGAGGC **R**: TCACTGGCGGAATCCAACAC	95ºC,2 min; 25 cycles (95 ºC, 30 s;59 ºC,30 s; 72 ºC, 50 s), 72 ºC,10 min	
***peg-344*** (411 bp)	**F**:AAAGGACAGAAAGCCAGTG **R**: CAATGACGAGGGGGATAATC	95ºC,2 min; 25 cycles (95 ºC, 30 s;53 ºC,30 s; 72 ºC, 30 s), 72 ºC,10 min	
***rmpA*** (332 bp)	**F**:GAGTAGTTAATAAATCAATAGCAAT **R**: CAGTAGGCATTGCAGCA	95ºC,2 min; 25 cycles (95 ºC, 30 s;50 ºC,30 s; 72 ºC, 40 s), 72 ºC,10 min	
***rmpA2*** (430 bp)	**F**: GTGCAATAAGGATGTTACATTA **R**: GGATGCCCTCCTCCTG		

### Identification of hvKp strains

#### Molecular identification of hvKp

Uniplex PCR was performed on *K. pneumoniae* isolates to detect virulence genes (*rmpA*, *rmpA2*, *iucA*, *iroB*, and *peg-344*), molecular biomarkers for hvKp [15]. The reaction was performed according to [15, 40] using 5 µl 2x EmeraldAmp MAX PCR Master Mix (Takara, Japan), 0.75 µl forward primer, 0.75 µl reverse primer (20 pmoles/µl), 1 µl genomic DNA (50 ng/µl), and 2.5 µl water. The sequences, annealing temperatures of primers, and amplicon sizes are listed in Table 1. Isolates that carry at least one of the biomarker virulence genes are considered hvKp.

#### Phenotypic confirmation of hvKp

The string test was applied to all isolates with any of the biomarker virulence genes. The test was conducted as previously described [41, 42].

### Statistical analysis

Statistical analysis was conducted in R (version 4.2.2, R Foundation for Statistical Computing). The strains were clustered using the pheatmap library (version 1.0.12) [43].

## Results

### Occurrence of *klebsiella pneumoniae* in oysters

Based on the culture and phenotypic characterization methods, 16 confirmed *K. pneumoniae* isolates were recovered from 33 fresh oyster pooled samples at different retail fish markets in Egypt with a 48.5% prevalence rate.

### Phenotypic and genotypic detection of carbapenem-resistant *K. pneumoniae* (CRKP)

The antibiogram profile of the confirmed *K. pneumoniae* isolates against the carbapenem antibiotics group, meropenem (10 µg) and ertapenem (10 µg), was assessed. Among the 16 *K. pneumoniae* isolates, thirteen were carbapenem-resistant, one was intermediate-resistant, and two were sensitive to it, as shown in Table 2.

**Table 2:** Phenotypic and genotypic carbapenem resistance profile of the confirmed *K. pneumoniae* isolates

Kp isolates	Antibiogram profile (10 µg)		Carbapenem resistance genes			
	MRP	ETP	*bla*_VIM_	*bla*_OXA−48_	*bla*_NDM_	*bla*_KPC_
**Kp.15**	R	R	**+**	**+**	**+**	**+**
**Kp.33**	R	R	**+**	**+**	**+**	**+**
**Kp.10**	R	R	**+**	**+**	**+**	**-**
**Kp.16**	R	R	**+**	**-**	**+**	**+**
**Kp.12**	R	R	**+**	**+**	**-**	**-**
**Kp.13**	R	R	**+**	**+**	**-**	**-**
**Kp.17**	R	R	**+**	**+**	**-**	**-**
**Kp.18**	R	R	**+**	**+**	**-**	**-**
**Kp.11**	R	R	**-**	**+**	**-**	**+**
**Kp.22**	I	I	**-**	**+**	**-**	**+**
**Kp.8**	R	R	**-**	**-**	**+**	**+**
**Kp.31**	R	R	**+**	**-**	**+**	**-**
**Kp.30**	R	R	**+**	**-**	**-**	**-**
**Kp.25**	R	R	**+**	**-**	**-**	**-**
**Kp.21**	S	S	**-**	**-**	**-**	**-**
**Kp.27**	S	S	**-**	**-**	**-**	**-**
**Total (%)**			**11 (68.8)**	**9(56.3)**	**6 (37.5)**	**6(37.5)**

R, I, and S, represent to Resistance, Intermediate resistance and sensitive isolates, respectively

All *K. pneumoniae* isolates in the current study were screened for carbapenemase-encoding genes (*bla*_VIM,_*bla*_OXA−48_, *bla*_NDM_, *bla*_KPC_). The results indicated that the *bla*_VIM_ gene was the most frequently detected gene, with a prevalence of (11/16; 68.8%), followed by the *bla*_OXA−48_ gene (9/16; 56.3%). Six isolates (37.5%) carried the other two genes, *bla*_NDM_ and *bla*_KPC_ (Table 2).

The genotypic profile of phenotypic carbapenem-resistant *K. pneumoniae* isolates (*n* = 13) and carbapenem-intermediate resistant *K. pneumoniae* isolates (*n* = 1) revealed that all isolates were carbapenemase producers. These isolates harbor carbapenemase-encoding genes (*bla*_VIM,_*bla*_OXA−48_, *bla*_NDM_, and *bla*_KPC_) either in combination or as a single gene. Conversely, the carbapenem-sensitive *K. pneumoniae* isolates (*n* = 2) were negative for all genes (Table 2).

### Genotypic and phenotypic identification of hvKp

The virulence genes *rmpA*, *rmpA2*, *iucA*, *iroB*, and *peg-344*, which are biomarkers for hvKp, were identified using molecular detection; ten isolates (10/16) yielded hvKp strains, with an isolation rate of 62.5%. Out of all the biomarker virulence genes identified, *peg-344* was the most prevalent one (6/10; 60%), followed by *rmpA2* (50%), *rmpA* (40%), and *iucA* (20%). Meanwhile, *iroB* was detected only in one isolate (10%). Six of the ten hvKp isolates showed multiple virulence genes. All four genes, *peg-344*, *rmpA*, *iucA*, and *iroB*, were detected in one isolate. The other five had different combinations of two genes: *rmpA*, *rmpA2*, *peg-344*, *or iucA*. One gene, *rmpA2* or *peg-344*, was identified in the remaining four isolates (Table 3).

**Table 3:** The genotypic profile of hypervirulent *K. pneumoniae* (hvKp) isolated from oysters in Egypt

HvKp isolates (*n* = 10)	Hypervirulence genes				
	*Peg-344*	*rmpA2*	*rmpA*	*iucA*	*iroB*
**Kp.18**	+	-	+	+	+
**Kp.12**	**-**	**+**	**+**	**-**	**-**
**Kp.16**	**-**	**+**	**+**	**-**	**-**
**Kp.8**	**+**	**-**	**-**	**+**	**-**
**Kp.15**	**+**	**+**	**-**	**-**	**-**
**Kp.31**	**+**	**-**	**+**	**-**	**-**
**Kp.10**	**+**	**-**	**-**	**-**	**-**
**Kp.13**	**-**	**+**	**-**	**-**	**-**
**Kp.17**	**-**	**+**	**-**	**-**	**-**
**Kp.30**	**+**	**-**	**-**	**-**	**-**
**Total (%)**	**6 (60)**	**5 (50)**	**4 (40)**	**2 (20)**	**1 (10)**

All isolates with any of the biomarker virulence genes were confirmed as hypervirulent via string test showing hypermucoviscous phenotype of colonies and formation of a viscous thread-like string more than 5 mm long on an agar plate.

### Coexistence of carbapenemases with hypervirulence genes in carbapenem-resistant hypervirulent *Klebsiella pneumoniae* (CR-hvKp)

In this study, all the hvKp isolates showed resistance to carbapenems, leading to the emergence of CR-hvKp. Furthermore, most of these isolates possessed more than one virulence gene combined with more carbapenemase-encoding genes (Kp15, Kp18, Kp16, Kp8, Kp12, and Kp31). Nevertheless, no virulence genes were present in carbapenem-intermediate resistant and carbapenem-sensitive isolates.

**Fig. 1:**
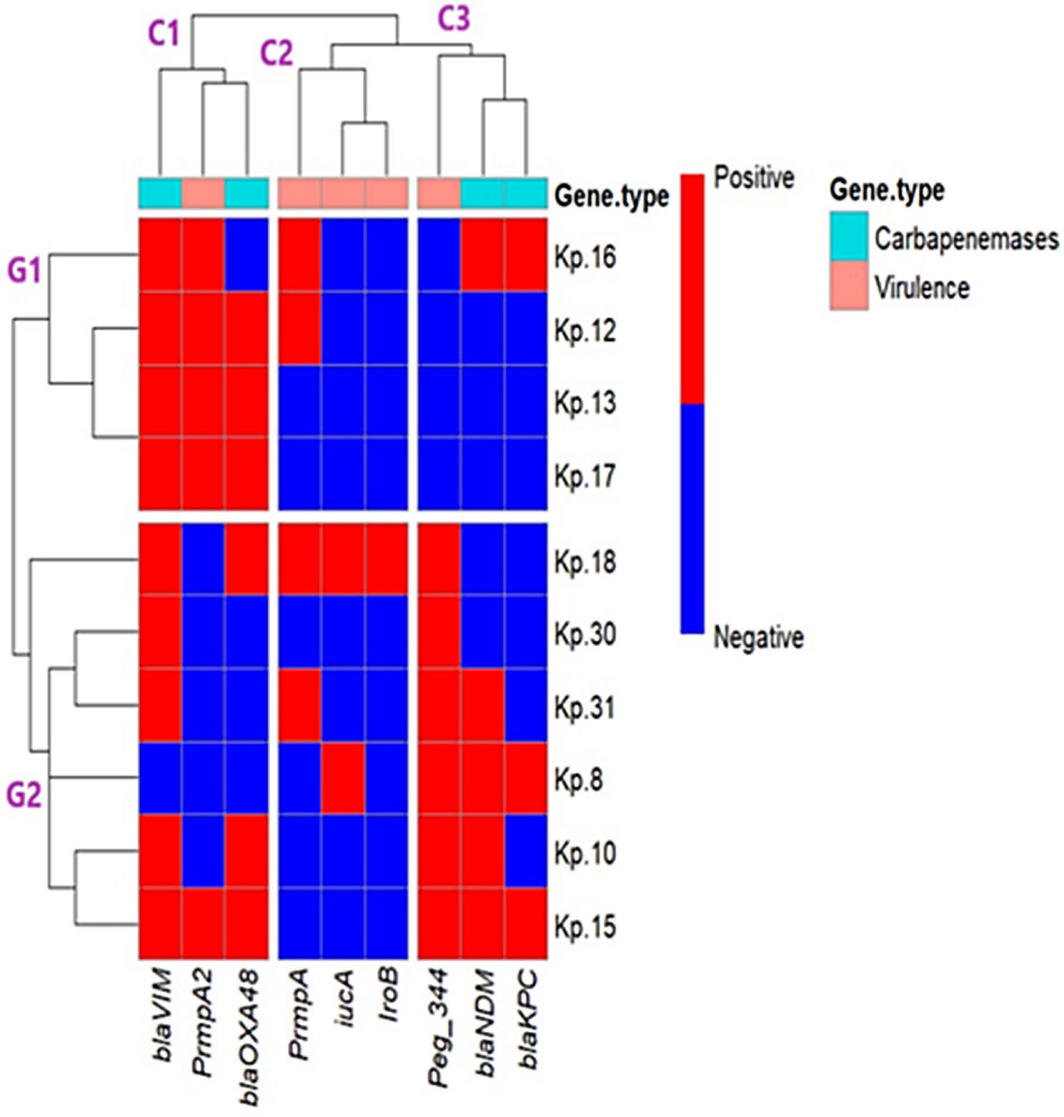
Heat map showing the Coexistence of carbapenemases with hypervirulence genes in carbapenem-resistant hypervirulent *Klebsiella pneumoniae* (CR-hvKp) (*n* = 10*)*

The heat map (Fig. 1) represents the grouping of CR-hvKp isolates (*n* = 10) into two main categories (G1 and G2) based on the presence of both carbapenem-resistant genes (*bla*_VIM,_*bla*_OXA−48_, *bla*_NDM_, and *bla*_KPC_) and hypervirulence genes (*rmpA, rmpA2, iucA, iroB*, and *peg-344*). The top of the heat map (C1, C2, and C3) illustrates the type of genes present.

Within this clustering, two CR-hvKp strains (Kp13 and Kp17) originating from the same retail fish market displayed identical profiles of *bla*_VIM_, *bla*_OXA−4*8*_, and *rmpA2*. Interestingly, they were found in the same clade with another isolate (Kp12), which shared the presence of *bla*_VIM_, *bla*_OXA−48_, *rmpA*, and *rmpA2*. In addition, (Kp18) was found to possess identical carbapenemases (*bla*_VIM_ and *bla*_OXA−48_) as well as multiple virulence genes (*rmpA, iucA, iroB*, and *peg-344*). Most CR-hvKp isolates had the *bla*_VIM_ gene, while only a few had the *bla*_KPC_ gene.

## Discussion

*Klebsiella pneumoniae* is one of the most frequently encountered pathogens that has attracted global research interest, as it causes life-threatening infections in humans and animals [44, 45]. In Egypt, Sabry et al. [46] reported that *K. pneumoniae* is a prevalent *Enterobacteriaceae* species among fish and fish vendors. Nevertheless, the role of aquatic environments, particularly oysters, in *K. pneumoniae* colonization and subsequent human infections remains limited.

In this study, the isolation rate of *K. pneumoniae* was 48.5%. This finding is supported by the results reported by Xu et al. [27], who detected a higher occurrence of *K. pneumoniae* (63.8%) in aquatic animals, particularly mollusks near the bottom. The presence of *K. pneumoniae* in mollusks raises substantial concerns regarding food safety and its potential impact on human health.

Aquatic environments have been identified as reservoirs for antibiotic resistance due to the improper use and excessive administration of antibiotics for disease management [47]. Recent reports have underscored the emergence of carbapenem-resistant *K. pneumoniae* (CRKP), even in marine bivalves, posing a disturbing trend in antibiotic resistance [29, 48]. Among the confirmed *K. pneumoniae* isolates tested against the carbapenem antibiotics group, two showed sensitivity, one exhibited intermediate resistance, and thirteen displayed carbapenem resistance. This trend of increasing resistance to carbapenems is consistent with data from the China Antimicrobial Surveillance Network (CHINET), which reported rising resistance rates of *K. pneumoniae* to imipenem and meropenem over the years from 3.0% to 2.9% in 2005 to 25% and 26.3% in 2018, respectively [34].

The genotypic analysis of all *K. pneumoniae* isolates revealed that those with phenotypic intermediate and resistant carbapenem profiles were carbapenemase producers. These isolates carried carbapenemase-encoding genes (*bla*_VIM_, *bla*_OXA−48_, *bla*_NDM_, and *bla*_KPC_) as individual genes or in various combinations. This finding underscores the crucial role of genetic factors in conferring carbapenem resistance and highlights the challenges in combating these resistant strains. This result can be attributed to the presence of these genes on plasmids, which can be transferred horizontally between different bacterial strains and species [49].

The present study highlighted the predominance of *bla*_VIM_ over the other genes *bla*_OXA−48_, *bla*_KPC_, and *bla*_NDM_, indicating that aquatic organisms could potentially serve as a reservoir for carbapenemase-producing *K. pneumoniae*. This finding aligns with previous studies that reported a high frequency of *bla*_VIM_ in CRKP isolates from human samples in Saudi Arabia [50] and Egypt [51]. Besides, Hamza et al. [52] reported a high prevalence of *bla*_KPC_ among *Enterobacteriaceae* isolates from integrated fish farms in Egypt. This result sheds light on the alarming prevalence of carbapenem-resistant *K. pneumoniae* in aquatic environments, particularly mollusks, and underscores the urgent need for enhanced surveillance and intervention strategies to address this growing threat to public health.

The emergence of hvKp raises significant concern within the global health community, given its heightened pathogenicity and virulence characteristics [53]. Our results showed that the overall prevalence of hvKp was 62.5%; this is consistent with the report of Su et al. [54], who detected a 59.1% prevalence of hvKp.

In the present study, all biomarker virulence genes were identified among the obtained hvKp isolates (*n* = 10), *peg-344* being the most prevalent, followed by *rmpA2*, *rmpA*, *iucA*, and *IroB* genes. This finding agrees with Bulger et al. [16], who reported that most hypervirulent *K. pneumoniae* clones carry virulence plasmids containing the putative metabolite transporter *peg-344*. Additionally, our isolates often had *rmpA2* and *rmpA* genes, a finding consistent with Emam et al. [55] and Mario et al. [40], who reported the presence of these genes in hvKp isolates at a high frequency. Moreover, Hsu et al. [56] and Khattab and Hager [57] conveyed that more than half (55–100%) of hvKp strains express at least one copy of *rmpA* or *rmpA*2 genes. This underscores the utility of *rmpA* gene as a common diagnostic marker for hypervirulence [58]. On the other hand, we reported a low prevalence of *iucA and IroB* genes among hvKp isolates. These findings agree with studies in Egypt that detected a low frequency of the aerobactin-encoding gene (*iucA*) [40, 59]. In the current study, 6 out of 10 hvKp isolates carried more than one virulence gene, indicating the possible violent nature of such strains and their public health consequences [60].

The emergence of superbug CR-hvKp strains in retail oysters in Egypt is particularly worrisome. All the hvKp isolates in this study showed resistance to carbapenems, leading to the emergence of CR-hvKp. Furthermore, most of these isolates possessed more than one virulence gene combined with more carbapenemase-encoding genes, which made them more resistant to antibiotics and more likely to cause severe infections in hospital and community settings. This agrees with the findings of Zhao et al. [61], who reported that most CR-hvKp strains isolated from patients admitted to the intensive care unit (ICU) had diverse resistance determinants and hypervirulence genes.

Notably, the VIM*-*producing CR-hvKp strains were predominant, suggesting that VIM was the primary mechanism of carbapenem resistance in hvKp strains. On the other hand, we observed that CR-hvKp strains that produce KPC were rare, which is consistent with Zhang et al. [62], Zhang et al. [63], Wei et al. [64], and Wei et al. [65], who found that CR-hvKp strains carrying the *bla*_KPC_ gene are limited.

To understand the diversity and evolution of this pathogen, we grouped the CR-hvKp strains by their carbapenemases and virulence genes using the pheatmap library. We observed that two CR-hvKp isolates were part of the same cluster, which could be attributed to the potential exchange of carbapenem resistance and hypervirulence genes among various strains and species of bacteria through plasmid-mediated horizontal gene transfer [49]. The finding of CR-hvKp strains that have both carbapenem resistance and hypervirulence features in oysters in Egypt is very significant, and it sheds light on the potential role of marine bivalves, especially oysters, in the epidemiology of CR-hvKp strains. Oysters accumulate such strains in their tissues, which can be transmitted to them from the water, the environment, and during their processing, suggesting the potential transmission of these strains to consumers via the food chain, constituting a significant public health crisis [66].

## Conclusion

The research highlights the significant contribution of marine bivalves, particularly oysters, in disseminating CRKP, hvKp, and CR-hvKp. Antibiotic resistance and virulence profile revealed that aquatic environments act as focal points for carbapenemases and hypervirulence genes, affirming the elevated pathogenicity of these bacterial strains. These findings stress the need for rigorous food safety measures, monitoring veterinary antibiotic usage, and collaborative efforts to safeguard public health, particularly for seafood consumers.

## Data Availability

No datasets were generated or analysed during the current study.

## Abbreviations

CR-hvKp: Carbapenem-resistant hypervirulent *Klebsiella pneumoniae*
CRKP: Carbapenem-resistant *K. pneumoniae*
HvKp: Hypervirulent *K. pneumoniae*
CKP: Classic *K. pneumoniae*
CRE: Carbapenemase-Producing *Enterobacteriaceae*
KPC: *K. pneumoniae* carbapenemases
MBL: metallo-β-lactamases
VIM: Verona integron-encoded metallo-Beta-lactamase
NDM: New Delhi metallo-beta-lactamase
*OXA-48*: Oxacillinase-48
*Peg344*: Putative transporter
*RmpA* and *RmpA2*: Regulators of the mucoid phenotype
*IucA*: Aerobactin siderophore biosynthesis
*IroB*: Salmochelin siderophore biosynthesis
IACUC: Institutional Animal Care and Use Committee
CLSI: Clinical and Laboratory Standards Institute
CHINET: China Antimicrobial Surveillance Network
ICU: Intensive care unit
